# Medical News and Miscellany

**Published:** 1892-02

**Authors:** 


### Medical News and Miscellany.

### Correction.

Statements and publication made by me in the *Texas Health Journal* or elsewhere, reflecting upon the character and reputation of Dr. F. E. Daniel, were made upon hearsay testimony that I at the time believed to be true. Subsequent developments and counter testimony, however, convinced me of their incorrectness. I further agree to publish this statement in the *Texas Health Journal*, of January, and agree that it shall be also published in DANIEL'S MEDICAL JOURNAL. I regret the circumstances that led to the unpleasant controversy between Dr. Daniel and myself and am willing to drop this matter forever.

(Signed) J. R. BRIGGS.

From Texas Health Journal.

"Warts," says Dr. Romer, of Austin, Texas, are cured by the fresh flowers of the common weed, mullein, rubbed smartly on the excrescence.—*Sanitary Era.*

Will some one kindly inform the JOURNAL who "Dr. Romer of Austin, Texas," is? We challenge the *Sanitary Era* to produce him, or authority for the above statement.

ERRATUM.—In the leading editorial the "H" has accidentally been left out of "anachronism."

Dr. Max Urwitz has removed from Houston to Yorktown, Texas.

Dr. J. A. White has removed from Prairie Plains to Huntsville, Texas.

Dr. J. H. Earp has removed from Gatesville, Texas, to Corsicana, Texas.

Dr. Jno. F. Starley has removed from Bastrop, Texas, to Marlow, Indian Territory.

Dr. S. F. King, of Bells, Texas, is attending the New York Polyclinic. On his return to Texas he will locate at Sherman.

Dr. Chas. Marchand, of the "Brevet Chemical Co.," N. Y., has removed his great Peroxide of Hydrogen Laboratory from 10 W. 4th St. to No. 28 Prince St.

A Great Woman.—Where is the Ovariotomist?—Dr. W. H. Seale reports to the JOURNAL the case of a woman of thirty-five years who is the mother of fifteen children.

Death of Mrs. Lott.—The JOURNAL is pained to announce the death of Mrs. Lott, wife of Dr. M. K. Lott, of Eagle Pass, Texas, in New Orleans, on the 25th of January, ult.

The Board of Regents of the Texas University, on the 21st of January (ult.), electde Prof. Clark Edwards, of Massachusetts, Assistant Professor of Biology, at a salary of $2,000 a year.

Dr. Strickler, in discussing Dr. Keating's paper before the Colorado Springs Medical Society, claims to have "had the good fortune to make 'post mortem' examinations on several persons now living."

Attention is called to the advertisement of the Gundry Home at Catonsville, Md. Dr. Gundry is endorsed by the ablest alienists and asylum superintendents in America. For particulars, address as per card.

Sir Dr. Morell McKenzie died in London, Feb. 3rd, of bronchitis. Dr. McKenzie became famous in connection with the treatment of the throat of the late Emperor Frederick—a son-in-law of Queen Victoria.

---

**The Y. M. C. A., of Texas**, have raised a large amount of money by subscription, and having obtained permission from the Board of Regents, will erect a $25,000 building in the University grounds at Austin.

---

**Physicians and Students** are requested to read carefully the advertisement of the Baltimore College of Physicians and Surgeons, and to send to Dr. Thomas Opie, the Dean, for a catalogue. Mention the JOURNAL.

---

**Dr. W. M. Burger** has removed from Claude to Cornelia, Armstrong county. He has entered a section of land away out in the frontier of civilization, and is laying the foundation, the JOURNAL hopes, of a big fortune—he deserves it.

---

**Dr. S. H. Stout**, of Cisco, had an unusually severe attack of the prevailing influenza, generally called "la grippe." He was attacked on Christmas day, and was only fairly convalescent when we heard from him on the 20th of January.

---

**Death of Dr. Stone.**—A letter from Health Officer Perkins, of Sabine Pass, announces the death of Dr. T. M. Stone, of Jasper, Texas. Dr. Stone was an old member of the Texas State Medical Association, and a physician of prominence, held in very high regard all who knew him. He died on the 18th of January.

---

**Married**, at San Antonio, Texas, February 3rd inst., at Paine Methodist Episcopal Church, South, Dr. R. F. Minnock, of Waco, to Miss Tenie Alberta Thomas, of San Antonio.

The JOURNAL acknowledges the courtesy of an invitation to the wedding, and extends hearty congratulations to the happy couple. The bride has captured one of the handsomest young Doctors of the State.

---

**Dr. Jno. B. Renshaw**, of Decatur, Texas, has been appointed Assistant Physician to the Southwest Texas State Lunatic Asylum at San Antonio. This is an excellent appointment. Dr. Renshaw brings testimonials of high social and professional standing, and is said to be a young physician of high order of ability. Decatur, Wise county, has thus furnished two asylum officers, Dr. White, Superintendent at Austin, being a native of Decatur, as well as Dr. Renshaw.

---

**Dr. P. W. Johns**, so well and favorably known throughout Texas—a long time resident at San Antonio—has removed to Hot Springs, Ark., and opened up an office for general practice. The JOURNAL learns, with much pleasure, that the Doctor has fully regained his health, which, from overwork and exposure, was greatly impaired. It takes this opportunity to commend Dr. Johns to the profession of Arkansas, and the people, as a first-class physician, and a man worthy in every way of confidence and esteem.

---

**The College of Physicians** of Philadelphia announces that the next award of the Alvarenga prize, being the income for one year of the bequest of the late Señor Alvarenga, and amounting to about one hundred and eighty dollars, will be made on July 14, 1892. Essays intended for competition may be upon any subject in medicine, and must be received by the Secretary of the College on or before May 1, 1892. It is a condition of competition that the successful essay or a copy of it shall remain in possession of the College.

---

**Doctors Will Disagree.**—The telegraph dispatches to the *Statesman* of Jan. 28th, ult., announce the occurrence of a deplorable tragedy in the town of Liberty, Texas, on that day. Dr. Jas. P. Cooke, an old and highly respected physician of that place, was shot and killed by Dr. J. O. Furlow. The JOURNAL is not in possession of the particulars of the killing, nor of the causes that led to it. Dr. Cooke was a graduate of the University of Maryland School of Medicine, of the class of 1858. Dr. Furlow was a graduate of the Medical Department of University of Louisville, class of 1875, and formerly resided at Shelbyville, Texas. He had not long been a resident of Liberty.

---

**New Orleans Polyclinic, Fifth Annual Report.**—The growth and prosperity of this enterprise has been as remarkable as it is gratifying to its many friends. The number of matriculants in 1891 was nearly four times as great as in 1888. The school is now in its own building, but the use of the wards of the Charity Hospital will not be curtailed or interfered with. The directors say: "The great advantage which the New Orleans

Polyclinic enjoys over other post graduate schools is the free use which can be made of the immense number of patients in the in-door and outdoor clinics of that famous institution."

The fifth annual session will open April 7, and close May 18, 1892.

---

**Desquamation Following La Grippe.**—Dr. S. H. Stout, of Cisco, in a letter to the JOURNAL, calls attention to extensive desquamation following the febrile stage of this disease, "the prevailing influenza." In one case "it was as great as usually occurs after measles." The Doctor says: "I am satisfied, after observation of numerous cases besides my own, that the desquamation of the epithelium is a frequent consequence of the disease, and the cause of deaths from its sequellæ, a fact I have not seen insisted on; it seems to have been overlooked by writers and lecturers." The Doctor bears testimony also to the extreme muscular weakness and languor, so often complained of, and says he can understand, since his personal experience, why the old and feeble so often succumb to it.

---

**At the stated meeting** of the Medical Society of the County of New York, on Monday, Jan. 25, 1892, the subject of discussion was the Epidemic of Influenza.

The discussion was opened by Dr. Janeway, and after addresses by Drs. Jackson, Draper, and Robinson, Dr. Francis Delafield addressed the Society on the Treatment of Influenza. He stated as follows: The treatment consisted of putting the patient to bed and seeing that he was well nursed and had proper diet, while the disease was running its course. It was possible, however, for the physician to interfere with advantage in the case of certain complications. Of all the remedies suggested for the treatment of Influenza and its complications, such as severe headache, or neuralgia pains, etc., he had found nothing so reliable as Phenacetine, in doses of five grains every two hours. The catarrhal throat trouble, which is often present, he had treated successfully with aconite or salicylate of soda, with a solution of cocaine for local applications.—*Medical Record.*

---

**The Very Latest Discovery.**—E. E. Furney, M. D., in an article on the Hygiene of Infant Life (*Weekly Medical Review*, Dec. 19th, 1891) announces a discovery that will be a revelation to those who have regarded mother's milk as the most eligible diet for infants. He says:

"Every woman who loves her offspring ought to be taught the truth that woman's milk is an unsafe food.

"Almost any cooked food, if cleanly prepared and administered, is safer than woman's milk."

Shades of Galen and Hippocrates, what are we coming to? Is the mammary gland to become rudimentary only? If the breast and ovaries are both to go, what will become of the woman of the future?—*Medical Age.*

Yes, what will become of the "mammy" (mammæ)? A Furney notion, really. Perhaps the doctor is prepared to Furney-sh a substitute for breast milk—some kind of "*ine*," which we may expect to see advertised soon.

---

The Peroxide of Hydrogen.—Of all the remedies which have come into use recently, there is none, perhaps, which has given more general satisfaction, or which meets a wider range of therapeutic indications than this. Dr. Chas. Marchand, Pres. of the Drevet Chemical Co., appreciating the importance of this remedy, and of having a pure and reliable article, has put upon the market a preparation which has pretty well driven all others from the field, so completely does it fill the requirements. In fact, when Peroxide of Hydrogen is mentioned, it is Marchand's that is meant. Specify it in your prescriptions. It is adapted to all suppurating surfaces; to all diseases of mucous membrane, and is especially useful in diphtheria—used by spray—and in gonorrhœal and syphilitic affections. "Its power to destroy germs and septic matters, with which it comes in contact," says Dr. Chas. Phillips, of Boston, "is unsurpassed by any other germicide or antiseptic, and it is perfectly harmless to living tissue." Mr. Marchand will be pleased to furnish sample, if the JOURNAL is mentioned. See announcement under "contents."

---

Dr. F. L. Sim, the popular and jovial editor of the *Memphis Medical Monthly*,—(who does not know Sim?—and—love him?)—having suffered many years with an eye, has at last had it "plucked" out. We admire his "pluck," and the plucky manner in which he takes the consequences. We do not know whether or not the eye "offended" him,—we are sure it could never have offended anyone else,—"so genial in its light, so kindly in its beam,"—but out it is, at any rate, and Sim none the worse for it, we are pleased to see by the January number of his journal. He there tells the particulars, and indulges in some pleasantry anent the subject of eyes in general and his eye in particular. He seems to be somewhat troubled as to the cosmetic effect of the loss, and intimates that he will get an artificial eye, which, should it wink at the wrong girl, may be eliminated. It was a "Capital eye" he lost; and though he may become a "taurine youth with a vitreous optic," or conclude to "go one eye on it," through life—he will be none the less the genial gentleman and the upright physician—dearly loved by all who know him.

---

**For the Grippe.**—The best antiseptic for influenza, or La Grippe, is sulphite of sodium, 5 grains, every two hours till all pain leaves the system. My prescription is:—

℞ Sodii Sulphit ....................... ʒiv.

Aqua ....................... f℥vi.

M. S.—A teaspoonful in as much water every two hours till all pain is relieved and the patient is well.—*J. A. Monell, M. D., in M. and S. Reporter.*

Gelsemium alone is an abortive for la grippe in its *milder form, when there is little or no fever.* A general feeling of malaise, with wandering pains in the head, eyes, and body. What might with propriety be called grippy sensations. Two or three five (5) drop doses of fluid extract gelsemium, repeated at intervals of two hours, will seldom fail to afford complete relief.

The distressing bronchial cough, with a sense of pain and oppression in the lungs and throat, which accompanies so many cases of influenza is soon relieved by mustard plaster on the chest and the following expectorant mixture:

℞ Muriate of Ammonia ....................... ʒii
Morphia Sulphate ....................... gr. iii
Spirit of Chloroform ....................... ℥i
Tr. Scillae ....................... ʒii
Syr. Senega ....................... ℥i
Sp. Rock Candy ....................... q. s. add ℥iv

Mix. A teaspoonful in water every two or three hours.—*M. and S. Reporter.*

---

**A Rare Opportunity.**—The *Chicago Post Graduate Medical College* will begin, on the 28th of March, prox, *a special course* of two weeks in medicine and surgery. Drs. Nicholas Senn, J. B. Hamilton, Belfield and Fenger, all eminent and distinguished as teachers, will conduct the surgical clinics, while Drs. Ethridge, the Dean of the Rush Medical College, with Bauga and Hernotin will have charge of the gynecological work. Drs. Fletcher Ingalls and M. B. Brown will conduct the throat clinic. Other distinguished clinicians will be connected with the course, and in every department the work will be carried along, the very latest phases of practice being exemplified and illustrated. Amongst them we may mention Futterer, whose work on bacteriology recently electrified Germany; Hotz and Colburn, whose eye work is unsurpassed for neatness of technique, and successful results. Those who go to this course must expect to work, if they would profit by the opportunity; it will be an all-day matter each day of the two weeks, the clinics beginning at 8:30 a. m., and closing at 10 p. m.

This will be the fourth of these special working courses. They have been well patronized, and are especially intended for earnest men, who desire to brush up, and whose time is limited; excellent for the general practitioner who cannot spare six or eight weeks from home. The eyes of the world are turned to Chicago now, and railroad travel is cheap. Dr. M. B. Brown will be remembered as an old Texas confrere, formerly of Galveston. He will make the visit pleasant and profitable for the Texans who avail themselves of this opportunity. Write to him.

---

"The Doctor's Reciprocity."—If there is any one thing which Texas did need worse than any other, it is a depot of surgical instruments and appliances. It is just out of the question to talk of every physician keeping on hand a complete or any way near complete equipment to meet every emergency; it would require a fortune to do so. Hence, occasions frequently arise when even the best equipped practitioners need a certain instrument or appliance, and when he needs it he needs it badly—and right away. Time, therefore, and distance, are important questions; there is not time to send even to the nearest supply house —St. Louis. Mr. Chase, of the Fort Worth Pharmacy Company, had his attention called to these facts at a recent meeting of the Texas Medical Association, and believing the doctors would *reciprocate*; would sustain him in a conscientious endeavor to accommodate them, has opened up a very large and complete stock of the best, latest, and most approved instruments, and keeps in stock every instrument and surgical device that any emergency might demand, and offers them at prices that compare with eastern wholesale houses. He informs the JOURNAL that the company have been able to fill *ninety-five per cent.* of orders, to date; and encouraged by the patronage of leading physicians all over the State they are determined to stock up to fill the remaining five per cent., so that the doctors may feel sure, when in a tight, of being accommodated *at once* by return mail. The JOURNAL really thinks the profession should show a hearty appreciation of this enterprise, by sending to the company for their instruments —great and small. A trial is all that is asked to convince the most fastidious. See advertisement and mention the JOURNAL.

North Texas Hospital for the Insane, at Terrell, Tex.—Seventh annual report of managers and superintendent, year ending October 31, 1891. Dr. John Preston, Superintendent.

Remaining in asylum November 1, 1890, total patients, 490. Admitted since, 371. Whole number under treatment for the year, 861.

Discharged, 198; died, 50; escaped, 2. Remaining, November 1, 1891, 606.

The admitted, 371, averages a little more than one patient per day for every day in the year.

Percentage of discharged to number admitted—53.3; to whole number treated—23.

A majority of the deaths occurred from chronic diseases, nineteen having died from tuberculosis in some form.

The per cent. of deaths to whole number treated is not put down in report, but it is 5.8, a larger ratio than usual, we believe, but still a small percentage.

The report shows a steady increase of patients, there being 116 more in hospital than at last report, the admissions over deaths and discharges being about as 3 to 2. At this rate it will not be long before additional room is needed—the constant evil—and, we suppose, meantime the excess of insane over accommodated will be kept in jails or workhouses, to our everlasting disgrace. It takes a long time to get an appropriation for a new asylum building, and a longer time to get the building ready. Why not buy a very large farm and colonize the chronic incurables, as suggested by Dr. White?

There are some interesting statistics in this report.

Of the 371 admitted, 99 were farmers and 86 were farmers' wives—a total of 185, or exactly one-half the number admitted. The next highest number was laborers, 71. Then comes farmers' daughters, 12, and female servants, 11.

What is there about farming that could produce fifty per cent. of the insanity in Texas? Adding the farmers' daughters, the total is over fifty per cent.

Farmers, of all the people of the world, are removed from the causes which are usually said to produce insanity; but, doubtless, this class constitute fully fifty per cent. of population. Even admitting this, it would seem that insanity is of as frequent occurrence in the rural districts, and amongst those who lead quiet lives and follow healthful occupations, as amongst others. Of the 371 admitted there were five physicians. We are not so much susprised at the occurrence of insanity amongst this class as amongst the farmers and laborers.

Of the causes, the largest number, 81, is ascribed to heredity; unknown, 105. The next highest, 42, to ill health; 25 to masturbation (all males) Religious excitement, 16; while to intemperance are only ascribed 9 cases (all males), and abuse of narcotics, 5. Of the form of insanity—142 were acute mania.

| | |
|---|---|
| Total expense for the year | $112,193 91 |
| Amount received from pay patients and sent to State Treasurer | 2,918 32 |
| Actual running expense | 95,226 04 |
| Cost, per capita, for the year | 170 00 |
| Farm products | 4,006 00 |
| Garden | 3,933 04 |
| Dairy products | 4,144 55 |
| Average of patients | 560 |

This is a good report and a creditable showing. Dr. Preston is to be congratulated upon it.

### The N. Y. Post Graduate Hospital (and Babies' Wards) Seventh Annual Report.

The above report for the year ending Sept. 15, 1891, shows that more patients have been treated than any previous year. 842 House patients have been treated, of which 286 were babies; 13,007 patients treated in Dispensary, 43,791 visits made to Dispensary.

| | |
|---|---|
| The total expense of conducting this great work (general hospital) was | $29,960.48 |
| Total receipts including 2 beds endowed | 27,037.80 |
| Showing a deficit (pd. by Post Grad. Med. College | $ 2,922.68 |
| Babies Wards:—Receipts (many beds endowed) | $7,101.61 |
| Disbursements | 6,563.34 |

Balance on hand to be used in furnishing $538.27.

This is a noble work and far-reaching in its beneficence. While affording high class medical and surgical skill and the best nursing to thousands of indigent people, the physicians from all over the United States who go to New York to take the post graduate inatruction, have the benefit of the clinics thus afforded. The "babies wards" is, especially, a model charity. There thousands of little waifs—the children of poor working women who could not possibly furnish them either the necessary attention, nursing, medicine or procure medical attendance are tenderly cared for. This affords to the profession a splendid field for studying the different phases of infantile disease. There is a ward at the top of the house called the "Sunshine" ward where the little convalescents are taken for the invigorating sunlight. A kind of a play ground we suppose.

Dr. A. M. Phelps, who has contributed so much to orthopœdic surgery, has charge of the child's ward for cripples. Here, deformed and crippled children are not only relieved of suffering, and improved in health and appearance, but are rendered able to make their way in life, who would otherwise become a burden on the State.

In this great work woman's charity and devotion are conspicuous,—the best ladies of the city take an active interest in it; large sums of money have been raised by them by subscription, donation, and by tableaux, etc., and many visit the hospital daily.

Many have "endowed beds;" or, support beds during the year. Miss Julia Cooper and Mrs. Francis Vinson each donated $1000. D. B. St. John gave the Hospital $10,000.

It is in connection with this Hospital that the Post Graduate Medical College is conducted.

---

### A "Promising" Subscriber.

———, JACK COUNTY, TEXAS, January 13th, 1891.

*F. E. Daniel, Austin, Texas:*

DEAR SIR:—Yours of Dec. 7th 1891 Recd and will say in reply, that I am Ondley in Arrears 2 years for Medical Journal. I Ordered the Journal Stopt Last Dec When my 2 Years Expired. I were then Fixing to Leave the State Which I did, and remained a way untill July, the Matter Slipped my Mind before I left or I Should have remited the Amount $4 then Dew. I have not recd the Journal Since, Inclosed find your Statemen Which I Think will Satisfy you that i Am Correct, if Satisfactorly I will Remit that a Mount Soon.

Very Respectfuly

————————, M. D.

[Alas, the poor publisher. Such is the experience of many.—EDITOR.]

